# HORNBILL: a phase I/IIa trial examining the safety, tolerability and early response of BI 764524 in patients with diabetic retinopathy and diabetic macular ischaemia—rationale, study design and protocol

**DOI:** 10.1186/s13063-022-06527-y

**Published:** 2022-08-17

**Authors:** Victor Chong, Quan Dong Nguyen, Yasir Sepah, Andrea Giani, Elizabeth Pearce

**Affiliations:** 1 UCL Institute of Ophthalmology, University College London, London, UK; 2 Byers Eye Institute, Stanford University School of Medicine, Palo Alto, CA USA; 3 Boehringer Ingelheim International GmbH, Ingelheim, Germany; 4 Boehringer Ingelheim Pharmaceuticals Inc., Ridgefield, CT USA

**Keywords:** Diabetic retinopathy, Diabetic macular ischaemia, Clinical trial, HORNBILL, OCTA, OCT

## Abstract

**Background:**

Diabetic macular ischaemia (DMI) is a complication of diabetic retinopathy that leads to irreversible vision loss. DMI is characterised by reduced retinal vessel density and enlargement of the foveal avascular zone (FAZ). Despite its clinical burden, there is no formal consensus on the definition of DMI, and no approved treatment. Semaphorin 3A (Sema3A) is an axonal guidance molecule that blocks revascularisation of the ischaemic retina. Sema3A modulation is therefore a promising mechanism of action for the treatment of ischaemic eye diseases. BI 764524 is an intravitreal anti-Sema3A ischaemia modulator agent.

**Methods:**

HORNBILL (NCT04424290) is a phase I/IIa trial comprising a non-randomised, open-label, single rising dose (SRD) part and a randomised, masked, sham-controlled multiple dose (MD) part to investigate the safety, tolerability and early biological response of ischaemia modulator BI 764524 in adults (≥18 years) with DMI. DMI will be defined using optical coherence tomography angiography (OCTA) as either any degree of disruption in the retinal vascularity (SRD) or a FAZ of ≥0.5 mm^2^ (MD). Subjects in the SRD part will receive 0.5, 1.0 or 2.5 mg of BI 764524; the maximum tolerated dose will then be used in the MD part. A minimum of 12 subjects will be enrolled into the SRD part; planned enrollment is 30 for the MD part. The primary endpoint of the SRD part is the number of subjects with dose-limiting adverse events (AEs) until day 8. The primary endpoint of the MD part is the number of subjects with drug-related AEs from baseline to end of study, and secondary endpoints include change from baseline in the size of the FAZ, best-corrected visual acuity and central retinal thickness.

**Discussion:**

DMI is a poorly defined condition with no treatment options. HORNBILL is the first clinical trial to assess a treatment for DMI and to use OCTA as a means to define and examine DMI. The OCTA data generated in this trial could form the basis of formal diagnostic criteria for DMI. Furthermore, the novel mechanism of action (Sema3A modulation) explored in this trial has the potential to revolutionise the treatment landscape for patients with DMI.

**Trial registration:**

ClinicalTrials.govNCT04424290; EudraCT 2019-004432-28. Registered on 9 June 2020

**Supplementary Information:**

The online version contains supplementary material available at 10.1186/s13063-022-06527-y.

## Administrative information

Note: the numbers in curly brackets in this protocol refer to SPIRIT checklist item numbers. The order of the items has been modified to group similar items (see http://www.equator-network.org/reporting-guidelines/spirit-2013-statement-defining-standard-protocol-items-for-clinical-trials/).

**Table:** 

Title {1}	HORNBILL: a phase I/IIa trial examining the safety, tolerability and early response of BI 764524 in patients with diabetic retinopathy and diabetic macular ischemia – rationale, study design and protocol
Trial registration {2a and 2b}	ClinicalTrials NCT No. NCT04424290 EudraCT No. 2019-004432-28
Protocol version {3}	c29487972-03 (version 3.0, 28/09/2021)
Funding {4}	This study was funded by Boehringer Ingelheim.
Author details {5a}	QDN and YS: Byers Eye Institute, Stanford University School of Medicine, Palo Alto, CA, USA; AG and EP: Boehringer Ingelheim International GmbH, Biberach, Germany; VC: Royal Free Hospital, London, UK
Name and contact information for the trial sponsor {5b}	Jaana Harjula Boehringer Ingelheim Finland Ky, Tammasaarenkatu 5, FIN - 00180 Helsinki, Finland; Phone +358 10 3102 847; Fax +358 10 3102 997
Role of sponsor {5c}	Boehringer Ingelheim was involved in the design of the study, in the collection, analysis, and interpretation of data, and in writing the manuscript.

## Introduction

### Background and rationale {6a}

Diabetic retinopathy (DR) is the most common microvascular complication of diabetes in the working-age population [1, 2]. Roughly one-third of all people with diabetes have concurrent DR [3, 4], and DR is the leading cause of vision loss in adults of working age (20–74 years) [3–8]. Diabetic macular ischaemia (DMI) is considered to be a common complication of DR. Estimates vary according to different definitions and imaging modalities, but up to 77% of people with DR are reported to have DMI, with the greatest prevalence reported in patients with proliferative DR [9–11].

DMI is characterised by decreased retinal vessel density and/or non-perfusion of the superficial vascular complex and deep vascular complex of the eye [12–16]. DMI is also associated with enlargement and disruption of the foveal avascular zone (FAZ); increased FAZ size correlates with reduced visual acuity [17–19]. Chronic ischaemia of the retinal tissue ultimately leads to photoreceptor death and neuronal loss [20], resulting in progressive and irreversible vision loss [9, 10]. DMI is frequently comorbid with other complications of DR, such as diabetic macular oedema (DME) [9]. As DME often causes segmentation artefacts in retinal images [21], it may confound the identification of specific disease characteristics (including DMI); it is therefore vital to develop methods to overcome this potential issue. However, optical coherence tomography angiography (OCTA) remains a promising and relatively new retinal imaging technique. As OCTA is non-invasive and allows the assessment of blood vessels in 3D, it has great potential both to identify and to provide detailed information on DMI as a clinical trial endpoint [22].

There is no approved treatment to prevent or slow the onset or progression of DMI. This means that physicians are often reticent to diagnose a subject with DMI, as they are unable to provide any treatment option. Furthermore, there is a lack of consensus among researchers and physicians on how to define DMI [22].

Semaphorin 3A (Sema3A) is an axonal guidance molecule that can inhibit angiogenesis by vasorepulsion of blood vessels [23, 24]; in the eye, Sema3A blocks revascularisation of the ischaemic retina [25, 26]. Sema3A is therefore a promising target for treating ischaemic eye diseases. BI 764524 is an intravitreal anti-Sema3A ischaemia modulator agent. Preclinical data show that BI 764524 reduces the area of ischaemia in mouse retinas with oxygen-induced retinopathy [27]. HORNBILL is the first clinical trial assessing a treatment for DMI and the first trial to use OCTA as a means to define and examine DMI.

### Objectives {7}

HORNBILL (NCT04424290) is a phase I/IIa trial comprising a non-randomised, open-label, single rising dose (SRD) part and a randomised, sham-controlled multiple dose (MD) part to investigate the safety, tolerability and early biological response of ischaemia modulator BI 764524 in subjects with DMI.

### Trial design {8}

The trial will be conducted in compliance with the approved protocol and the ethical principles laid down in the Declaration of Helsinki and in accordance with the International Conference on Harmonization Harmonized Guideline for Good Clinical Practice, relevant Boehringer Ingelheim (BI) standard operating procedures, EU regulation 536/2014 and local regulations.

**Fig. 1:**
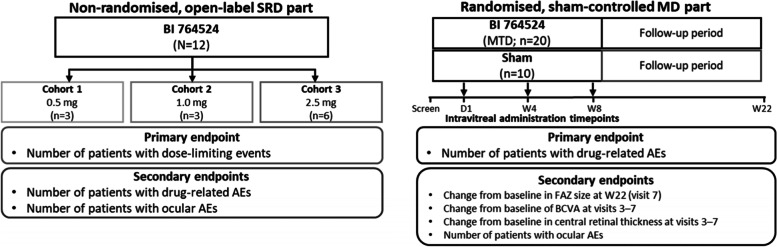
HORNBILL trial design. AE, adverse event; BCVA, best-corrected visual acuity; D, day; FAZ, foveal avascular zone; MD, multiple dose; MTD, maximum tolerated dose; SRD, single rising dose; W, week

The SRD part is non-randomised, open label and will not include a control (Fig. 1). Three dosing cohorts are planned: 0.5 mg, 1.0 mg and 2.5 mg of intravitreal BI 764524. A minimum of 12 subjects will be enrolled, three each to the 0.5 mg and 1.0 mg cohorts, and six to the 2.5 mg cohort. The MD part will only begin if the highest dose (2.5 mg) is tolerated. Following baseline vision and OCTA assessment, subjects in the MD part will be randomised. The MD part will be partially masked and sham controlled (Fig. 1). Two cohorts are planned: one cohort receiving the maximum feasible dose of BI 764524 as determined in the SRD part and one sham cohort. In total, 30 subjects will be enrolled, 20 to the dosed cohort and 10 to the sham cohort.

## Methods: participants, interventions and outcomes

### Study setting {9}

The trial will be conducted in two countries (US and UK) across approximately 20 locations that have specialist retinal or diabetes centres. A full list of study centres can be found on the clinical trials website (NCT04424290).

### Eligibility criteria {10}

Subjects who are ≥18 years of age with proliferative diabetic retinopathy and evidence of DMI who have been treated with pan-retinal photocoagulation are eligible for inclusion. DMI is defined in the SRD part as the presence of any degree of disruption in retinal vascularity within the superficial and/or deep retinal plexus, per investigator discretion (using OCTA). In the MD part, DMI is defined as a FAZ of ≥0.5 mm^2^; if the FAZ is <0.5 mm^2^, an enlarged peri-foveal inter-capillary space in at least one quadrant is deemed acceptable for inclusion.

In the SRD part, inclusion criteria will comprise the presence of DMI per investigator assessment, a best-corrected visual acuity (BCVA) of ≤55 letters and HbA1c of ≤12.0%. Subjects will be excluded if they have additional eye disease in the study eye that could compromise BCVA (such as uncontrolled glaucoma, age-related macular degeneration, ischaemic optic neuropathy, retinal vein occlusion, vitreomacular traction or aphakia), prior intraocular surgery within 3 months of screening, active intraocular inflammation in the study eye, active infectious conjunctivitis in either eye, or anterior segment or vitreous abnormalities that would preclude adequate imaging, or intravitreal anti-vascular endothelial growth factor (VEGF) injections, intravitreal steroids or treatment with a macular laser within 3 months of enrolment. In addition, in the MD part, inclusion criteria will comprise a BCVA of ≤85 letters. Subjects will be excluded if they have DME, disruption of the retinal inner layers (DRIL), epiretinal membrane, heavily lasered macula or prior vitrectomy.

### Who will take informed consent? {26a}

Receipt of signed and dated informed consent is a requirement for inclusion in the trial. A trial investigator or delegate will obtain informed, freely given, written consent from each subject via a consent form, after confirming that the subject understands the content of the form. The investigator or his delegate will then sign and date the consent form. If a trial collaborator has given a supplementary explanation to the subject, the trial collaborator will also sign and date the consent form.

### Additional consent provisions for collection and use of participant data and biological specimens {26b}

On the consent form, participants will be asked if they agree to the use of their data should they choose to withdraw from the trial. Participants will also be asked for permission for the research team to share relevant data with people from the centres taking part in the research or from regulatory authorities, where relevant. This trial does not involve collecting biological specimens for storage and future use.

### Interventions

#### Explanation for choice of comparators {6b}

As the SRD part is exploratory, there will be no comparator. In the MD part, a sham injection will be used as a comparator. This study was designed with the goal of minimising the number of subjects exposed to sub-therapeutic doses, while preserving safety and ensuring rapid dose finding.

#### Intervention description {11a}

Dose selection in this study is based on the pharmacokinetics (PK) of BI 764524 in rabbits, which is similar to that of bevacizumab, and therefore expected to be similar to the PK of bevacizumab in humans; the planned dosing cohorts are 0.5 mg, 1.0 mg and 2.5 mg of intravitreal BI 764524. A Bayesian logistic regression model (BLRM) employing the escalation with overdose control principle (EWOC) will be used for guiding the dose escalation [28, 29]. If no dose-limiting events have been observed for at minimum the 7-day evaluation period, the BLRM will be updated with the newly accumulated data. Escalation will be permitted if the EWOC criteria are met. Treatment will occur on the first visit after the screening period, with a follow-up of 14 weeks (Table 1). Each subject will receive a single intravitreal dose of BI 764524. The drug will be administered into the subject’s worse-seeing eye (assessed by BCVA) to further minimise the risk of any negative impact of treatment on the subject’s vision. In cases where the BCVA is equal between eyes, the choice of eye is at the principal investigator’s discretion.

**Table 1:**
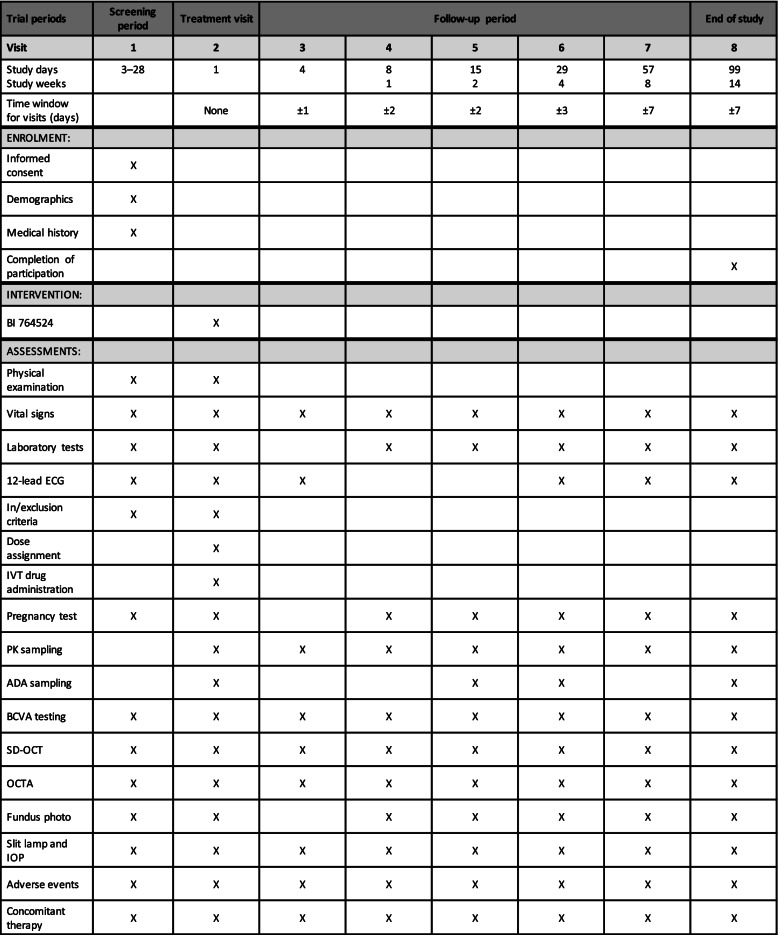
Endpoint measures and timings for the SRD part

*ADA* anti-drug antibody, *BCVA* best-corrected visual acuity, *ECG* electrocardiogram, *IOP*, intraocular pressure, *IVT* intravitreal, *OCTA* optical coherence tomography angiography, *PK* pharmacokinetic, *SD-OCT* spectral-domain optical coherence tomography angiography

In the MD part, each subject will receive three intravitreal or sham doses every 4 weeks over an 8-week period, with an additional 14-week follow-up (total study time of 22 weeks; Table 2). For the sham injection, the hub of a syringe without a needle is pressed against the conjunctival surface to simulate an actual injection. All pre- and post-injection procedures are otherwise identical between the MD part cohorts. As the personnel of the trial centre will be masked, an unmasked pharmacist will prepare the intravitreal injection for administration, and an unmasked qualified physician with no other involvement in the trial will administer intravitreal/sham injections.

**Table 2:**
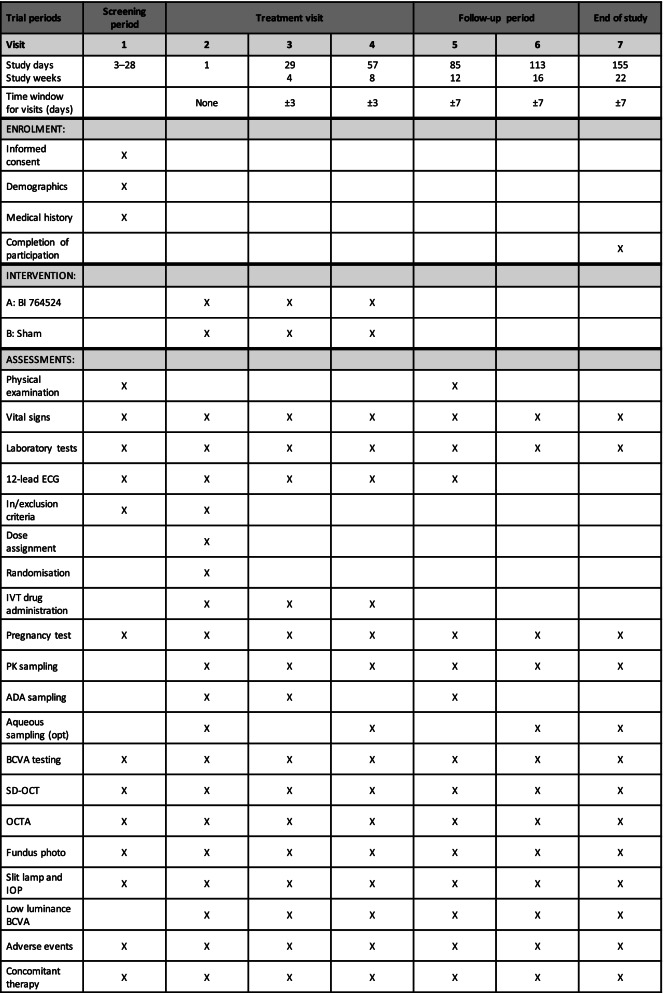
Endpoint measures and timings for the MD part

*ADA* anti-drug antibody, *BCVA* best-corrected visual acuity, *ECG* electrocardiogram, *IOP* intraocular pressure, *IVT* intravitreal, *OCTA* optical coherence tomography angiography, *PK* pharmacokinetic, *SD-OCT* spectral-domain optical coherence tomography angiography

#### Criteria for discontinuing or modifying allocated interventions {11b}

Subjects may withdraw from trial treatment without any justification. They may also discontinue if they are repeatedly non-compliant with important trial procedures, need to take concomitant medication that interferes with trial treatment, can no longer receive treatment due to medical reasons or have an adverse event (AE) or clinically significant laboratory change/abnormality that warrants discontinuation of treatment per investigator judgement. In the MD part, if treatment is discontinued, the subject will remain in the trial (with consent) and undergo the procedures outlined in Table 2. The trial as a whole will be discontinued if evidence emerges of efficacy/safety information that invalidates the positive benefit–risk assessment delineated prior to the trial start. This would include the emergence of one serious AE (SAE) in a single subject, or two severe AEs in separate subjects, that are confirmed as having a causal relationship with treatment administration.

#### Strategies to improve adherence to interventions {11c}

All trial medication will be administered by a qualified physician at the trial study centre, which will ensure subject adherence to the trial protocol.

#### Relevant concomitant care permitted or prohibited during the trial {11d}

For the study eye, no additional treatment of any kind will be allowed during the trial. The fellow eye can receive any on-label drug; however, any intravitreal treatment or use of medications known to be toxic to the retina, lens or optic nerve (e.g. deferoxamine, chloroquine/hydroxychloroquine, chlorpromazine, phenothiazines, tamoxifen, nicotinic acid and ethambutol) is restricted during the trial.

#### Provisions for post-trial care {30}

There are no provisions planned for post-trial care.

### Outcomes {12}

The primary endpoint of the SRD part is the number of subjects with dose-limiting events from drug administration to day 8 (7 days post treatment). Secondary endpoints are the number of subjects with drug-related or ocular AEs from drug administration until end of study. Further endpoints include the change from baseline in BCVA, central retinal thickness and size of the FAZ in the superficial and deep retinal plexus at each visit, measurements of the systemic PK of BI 764524 and systemic immunogenic response. The primary endpoint of the MD part is the number of subjects with drug-related AEs from drug administration until end of study (week 22). Secondary endpoints include change from baseline in size of the FAZ in the superficial and deep retinal plexus at week 22 (visit 7, assessed by OCTA) and change from baseline in BCVA and central retinal thickness (assessed by spectral domain optical coherence tomography [SD-OCT]) at visits 3 to 7. Further endpoints include systemic exposure of BI 764524 after multiple doses, changes in low-luminance BCVA at visits 3 to 7 and the systemic immunogenic response. Safety and early biological response will be examined up to 14 weeks after the last injection.

### Participant timeline {13}

The participant timeline is shown in Tables 1 and 2.

### Sample size {14}

No formal statistical power calculations to determine sample size will be performed for the SRD part as it is an explorative phase. Based on the simulation study conducted to evaluate operating characteristics of the BLRM, about 15 evaluable subjects are expected to be treated in the dose-escalation part for the model to ensure reasonable operating characteristics relating to the maximum feasible dose recommendation. Sample size was calculated for the MD part based on the estimated likelihood of observing a treatment effect given the mean intraindividual variability of FAZ estimates, which have been previously derived [30]. Assuming a mean treatment difference of 10%, with 20 subjects in the BI 764524 arm and 10 subjects in the sham arm, the likelihood of observing a treatment effect larger than 6% (reduction in size of the FAZ of 0.0462 mm^2^) is 81.7%, and the likelihood of observing a treatment effect smaller than 4% (reduction in size of the FAZ of 0.0308 mm^2^) is 10.2%. With an assumed mean treatment difference of 0%, the likelihood of observing a treatment effect larger than 6% would be 12.3%, and the likelihood of observing a treatment effect smaller than 4% is 78.7%.

### Recruitment {15}

Potential participants will be identified by ophthalmologists at participating centres. The precise process for inviting patients will vary by local centre practice, but typically will include a physician referral letter or pocket card of eligibility criteria. The use of incentives will depend on local regulations, but all sites will be reimbursed where necessary according to fair market value while participants will be reimbursed according to local practices as approved by the independent ethics committees (IECs)/institutional review boards (IRBs).

## Assignment of interventions: allocation

### Sequence generation {16a}

No randomisation for the SRD part is required as all subjects within a cohort will receive the same treatment. For the MD part, the randomisation list will be generated using a validated system (Interactive Response Technology [IRT]) that uses a pseudorandom number generator and a supplied seed number; the resulting allocation is both reproducible and non-predictable. No stratification factors were used.

### Concealment mechanism {16b}

All subjects (SRD and MD part) screened must be registered with IRT. In the MD part, subjects will be randomised to receive either active treatment or sham injection according to a randomisation plan at visit 2 via IRT. The IRT is used to conceal the sequence until intervention assignment.

### Implementation {16c}

In the MD part, each centre will use the IRT system to randomise subjects. The allocation sequence is based on a randomisation list generated by the trial sponsor, and treatment assignment is controlled by the IRT system.

## Assignment of interventions: masking

### Who will be masked? {17a}

The SRD part will be open label and unmasked, with all participants receiving BI 764524. In the MD part, all trial centre personnel and subjects will be masked, but an unmasked qualified physician who is otherwise not involved in the trial will administer the intravitreal and sham injections. Sham injections are standard as a control for intravitreal injection. Prior research indicates that subjects are successfully masked in instances where only one eye receives either treatment or sham injection [31]. Within the central laboratory, readers involved with interval measurements will be masked with respect to treatment, visit and demographic information. At the sponsor centre, all trial data will be handled open label; this means that the clinical monitor, data manager, statistician, bioanalyst, pharmacokineticist, pharmacometrician and drug metabolism scientist, among others, will be unmasked. Single-masked design has been shown to reduce the effect of bias [32]; the study procedures ensure that the investigator’s knowledge of the next treatment will not influence the decision to enter a subject.

### Procedure for unmasking if needed {17b}

Emergency unmasking will be available to the investigator via the IRT system and will only be used in an emergency situation when the identity of the trial drug must be known by the investigator to provide appropriate medical treatment or otherwise assure safety of trial participants.

## Data collection and management

### Plans for assessment and collection of outcomes {18a}

For all endpoints, baseline will be defined as the value collected at visit 2 (Tables 1 and 2). All ophthalmic examinations will be performed on both eyes. All assessments will be performed by a qualified person, and only specified OCT and OCTA equipment will be used. OCT and OCTA images will be sent to an independent central reading centre for evaluation; a detailed manual for OCT image acquisition and data transmission will be provided. The change in baseline FAZ size in mm^2^ will be assessed by OCTA at each visit in the SRD and MD parts (Tables 1 and 2). Patients will be refracted before assessing BCVA, according to the instructions in the manual provided Additional file 1. Following refraction, BCVA will be assessed using the ETDRS visual acuity letter chart at a distance of 4 m (ETDRS Chart 1 is used for the right eye, and ETDRS Chart 2 for the left eye), according to the instructions in the manual provided. The assessment will be performed by a trained person under pre-specified standardised conditions. Low-luminance BCVA will be measured using a mesopic filter and the ETDRS visual acuity letter chart, following the method described in the manual provided.

### Plans to promote participant retention and complete follow-up {18b}

The coordinating investigator will work with trial sponsors and investigators to ensure subject enrolment and retention. Measures to minimise subject withdrawal rate include careful subject selection, appropriate explanation of the trial requirements and procedures prior to first administration of trial medication and explanation of the consequences of withdrawal from the trial. If subjects wish to withdraw from the trial, they can either stop trial medication and participation completely or stop trial medication but continue to participate in trial visits.

### Data management {19}

Data management will be conducted in accordance with BI standard operating procedures. BI’s data management process supports International Council for Harmonisation of Technical Requirements for Pharmaceuticals for Human Use Good Clinical Practice. Data are collected directly via either an electronic case report form or via external data upload, for example for central laboratory results. In accordance with regulatory requirements, the centre trial investigator will prepare and maintain source documents and trial records for each trial patient, including all observations and other data pertinent to the investigation. Originals or copies of laboratory results and other imaging or testing results (with proper documented medical evaluation, in validated electronic format, if available) will be electronically transferred and uploaded into the trial database. Coding is done according to the current available dictionaries, with updates twice per year: range checks are established during database set-up. The Medidata Rave Electronic Data Capture system will be used for database set up and conduct. The investigator/institution will allow trial-related monitoring, audits, IRB/IEC review and regulatory inspections. Direct access will be provided to all source documents/data, including progress notes and copies of laboratory and medical test results, which must be available at all times for review by the clinical research associate, auditor and regulatory inspector.

All data management procedures are documented in the trial Data Management Plan (DMP), which describes the processes and procedures for the collection, processing and quality control of clinical trial data performed by Data Management throughout the lifecycle of the clinical trial; these processes and procedures are implemented to create and maintain high-quality data collection systems and to ensure data integrity. The DMP serves as a communication and reference tool for clinical trial teams to create and maintain a high-quality database ready for analysis.

### Confidentiality {27}

Data protection and data security measures are implemented for the collection, storage and processing of patient data in accordance with the principles 6 and 12 of the WHO GCP handbook. Participants’ data will be collected via a secure electronic data capture system. System access rights are controlled by BI, and dataset access is limited to BI. BI endorses the Principles for Responsible Clinical Data Sharing set out by Pharmaceutical Research and Manufacturers of America and The European Federation of Pharmaceutical Industries and Associations. BI provides redacted clinical study reports and clinical documents on request. Furthermore, BI provides qualified scientific and medical researchers with access to de-identified, analysable, patient-level clinical study data. Further information may be found on BI’s Data Transparency website (https://www.mystudywindow.com/).

Individual patient data obtained as a result of this trial are considered confidential and disclosure to third parties is prohibited with the following exception: personalised treatment data may be given to the patient’s personal physician or to other appropriate medical personnel responsible for the patient’s welfare. Data generated at the site as a result of the trial will be available for inspection on request by the participating physicians, the sponsor’s representatives, the IRB/IEC and regulatory authorities.

Before providing any copy of subject source documents to the sponsor, the investigator will ensure that all subject identifiers (e.g. name, initials, address, phone number and social security number) have been removed or redacted.

### Plans for collection, laboratory evaluation and storage of biological specimens for genetic or molecular analysis in this trial/future use {33}

Per item 26b, there will be no biological specimens stored for future use in this trial.

## Statistical methods

### Statistical methods for primary and secondary outcomes {20a}

No testing of statistical hypotheses is planned. Data will be analysed using a generalised mixed linear model. The assumed treatment effect of BI 764524 compared with sham treatment after 3 months of treatment is 10%. Change in central retinal thickness (mm) will be assessed by SD-OCT at each visit in the SRD and MD parts (Tables 1 and 2). Descriptive statistics will be computed to describe the empirical distributions and descriptive *p*-values will be calculated. The change from baseline in thickness will be analysed using a generalised mixed linear model. Descriptive statistics will be provided for each endpoint. Once the trial is complete, the statistical analysis plan will be available as part of the trial report package.

### Interim analyses {21b}

No inferential interim statistical analysis is planned. A preliminary analysis of PK parameters (area under the curve [AUC], C_max_), provided as individual values and geometric means of each cohort per dose level, may be performed, if feasible.

### Methods for additional analyses (e.g. subgroup analyses) {20b}

At the time of writing, no additional analyses are planned.

### Methods in analysis to handle protocol non-adherence and any statistical methods to handle missing data {20c}

There are no specific methods to handle protocol non-adherence or missing data.

### Plans to give access to the full protocol, participant-level data and statistical code {31c}

The current trial protocol has been shared in this manuscript. No patient-level data will be shared to third parties while the trial is ongoing.

## Oversight and monitoring {21}

### Composition of coordinating Centre and trial steering committee {5d}

For this trial, there will be no steering committee or coordinating centre. However, the coordinating investigator works with the sponsors and investigators to ensure subject enrolment and retention.

### Composition of data monitoring committee, its role and reporting structure {21a}

An extended safety monitoring committee (SMC) has been established for both SRD and MD parts. The SMC operates under the principles specified in the SMC charter. The SMC is a multidisciplinary group composed of the co-ordinating investigator, select participating investigators, members of the BI trial team and two experts independent from the BI trial/project team in the field of ophthalmology. SMC members are selected for their expertise in ophthalmology, their knowledge of the management of subjects with DMI and their experience in clinical trials and SMC activities. Details of the SMC responsibilities and procedures are described in the pre-specified SMC charter. The primary responsibility of the SMC is to ensure and protect the safety and well-being of the subjects participating in the trial. The primary objective of the SMC is to make a joint decision on both the dose escalation in the SRD part, and on the transfer to the MD part after the SRD part has been completed.

### Adverse event reporting and harms {22}

The investigator must report serious AEs, AEs of special interest (AESIs) and non-SAEs that are relevant for the reported SAE or AESI on the BI SAE form immediately (within 24 h) to the sponsor’s unique entry point. The same timeline will apply if follow-up information becomes available.

### Frequency and plans for auditing trial conduct {23}

Should an audit or inspection of this trial be conducted, the quality assurance auditor will have access to all medical records, the investigator’s trial-related files and correspondence and the informed consent documentation of this clinical trial. During the SRD part, the SMC will meet after each cohort has been dosed to review the patient data and make a recommendation about whether to open the next dose cohorts. Once the SRD part is complete, the SMC will meet to make a recommendation about transfer to the MD part of the trial. In the MD part, the SMC will meet after 10, 20 and 30 subjects have completed visit 5. Internal BI project management meet as needed according to the communications plans.

### Plans for communicating important protocol amendments to relevant parties {25}

Important protocol amendments will be listed on the trial’s clinicaltrials.gov webpage, and via the BlueSky training portal, clinical trial managers and the BI Clinical Trial portal ‘Clinerigize’. Any deviations from the protocol will be fully documented using a breach report form. The principal investigator is responsible for EC/IRB submissions according to the local regulations.

## Dissemination plans {31a}

The results of this trial will be disseminated at relevant congresses as interim data are available and as a full formal publication after the last subject has finished treatment and analyses have been completed. A plain language summary of the study results will be developed by BI and made freely available on the MyStudyWindow website (https://www.mystudywindow.com/).

## Discussion

The HORNBILL study is the first clinical trial of a potential treatment for DMI, and the first study to formally evaluate DMI. Despite being considered a common complication of DR, DMI is a poorly defined condition and leads to irreversible vision loss. There is no approved treatment and no consensus definition for this debilitating complication of DR, resulting in a large unmet need in the patient population. BI 764524, an anti-Sema3A agent, may be an effective treatment for retinal ischaemia. Sema3A modulation is a promising mechanism of action that has been shown to reduce ischaemia in a mouse model of oxygen-induced retinopathy. Sema3A blocks revascularisation of the ischaemic retina [25, 26]. Silencing expression of *Sema3A* in a mouse model of oxygen-induced retinopathy has been shown to maintain neuroretinal function [33]. Furthermore, recent data show that administration of the anti-Sema3A agent BI 764524 in a mouse model of oxygen-induced retinopathy significantly reduces avascular area size [27] and had a beneficial effect on intraretinal oedema and ischaemia in a retinal vein occlusion mouse model [34]. This suggests that Sema3A plays a targetable and pathological role in retinal ischaemia by contributing to vascular and subsequently neuronal damage, ultimately resulting in progressive vision loss. The current standard of care for non-proliferative diabetic retinopathy and DME is treatment with anti-VEGFs. However, DMI is not improved by anti-VEGFs and may even reduce treatment efficacy [35]. A novel mechanism of action (Sema3A modulation) that specifically improves ischaemia has the potential to revolutionise treatment of subjects with DR who experience ongoing vision loss despite responding well to the current standard of care, and after resolution of complications such as DME.

One challenge in developing a treatment for DMI is the current lack of a consensus definition. As the prevalence of DMI is not well described, the first SRD part of the study will use a less specific definition of DMI (any disruption in the retinal vascularity). This will ensure sufficient subject recruitment. In the second MD part, we will define DMI primarily by one of its hallmark characteristics, FAZ size [17–19]. This will ensure a high likelihood of excluding subjects without macular ischaemia, meaning that the study results will remain informative should a formal consensus definition be developed. The initial OCT and OCTA data generated in this trial could form the basis of formal diagnostic criteria for DMI.

To assess the efficacy of a treatment, it is vital to identify clinically meaningful anatomical and functional endpoints. FAZ size and central retinal thickness over time are associated with the anatomical progression of DR. Deep thickness measurements of the FAZ have been directly associated with changes in visual acuity; however, superficial OCTA measurements are less affected by imaging artefacts, particularly in the presence of known DMI comorbidities such as non-central DME [36–38]. We opted to use combined complex (superficial and deep) measurements of the FAZ in order to minimise image corruption and maximise our ability to accurately identify the characteristics of DMI. In conjunction with these anatomical measures, we will use functional measures of BCVA and low-luminance BCVA. Although change in BCVA is the most commonly used method for assessing visual function [14], evidence has shown that low-luminance BCVA is predictive of future vision loss in other retinal indications, such as geographic atrophy [39]. At present, there are no validated endpoints for use in clinical trials of DMI [22]; our results will support an informative set of standardised endpoints for use in future trials.

## Trial status

The current clinical trial protocol number is c29487972-05 (version 5.0, 20/04/2022). The first subject was enrolled on 20 July 2020, and the study will be completed by approximately October 2022. Currently, 12 subjects have been enrolled in the SRD part, which has completed as of 29 September 2021, and as of 28 June 2022 22 patients have been randomised to the MD part.

## Supplementary Information

**Additional file 1.**

## Data Availability

To ensure independent interpretation of clinical study results and enable authors to fulfil their role and obligations under the ICMJE criteria, Boehringer Ingelheim grants all external authors access to relevant clinical study data. In adherence with the Boehringer Ingelheim Policy on Transparency and Publication of Clinical Study Data, scientific and medical researchers can request access to clinical study data after publication of the primary manuscript in a peer-reviewed journal, regulatory activities are complete and other criteria are met. Researchers should use the link https://vivli.org/ to request access to study data and visit https://www.mystudywindow.com/msw/datasharing for further information.

## Abbreviations

AE: Adverse event
AESI: Adverse event of special interest
AUC: Area under the curve
BCVA: Best-corrected visual acuity
BI: Boehringer Ingelheim
BLRM: Bayesian logistic regression model
DME: Diabetic macular oedema
DMI: Diabetic macular ischaemia
DR: Diabetic retinopathy
DRIL: Disruption of the retinal inner layers
ETDRS: Early treatment diabetic retinopathy study
EWOC: Escalation with overdose control principle
FAZ: Foveal avascular zone
IEC: Independent ethics committee
IRB: Institutional review board
MD: Multiple dose
OCT: Optical coherence tomography
OCTA: Optical coherence tomography angiography
PK: Pharmacokinetics
SAE: Serious adverse event
SD-OCT: Spectral-domain optical coherence tomography
Sema3a: Semaphorin 3A
SMC: Safety monitoring committee
SRD: Single rising dose
VEGF: Vascular endothelial growth factor
