# Supplementary material for: HORNBILL: a phase I/IIa trial examining the safety, tolerability and early response of BI 764524 in patients with diabetic retinopathy and diabetic macular ischaemia—rationale, study design and protocol
Source: Trials. 2022 Aug 17;23:669. doi: 10.1186/s13063-022-06527-y (PMC9386971; doi:10.1186/s13063-022-06527-y)
Supplement: Supplementary file 1 — Additional file 1. [file 13063_2022_6527_MOESM1_ESM.docx]

**BI trials**

**Document: Refraction and BCVA Manual**

**Version: 1.0**

**Date: 23 Nov 2020**

Table of Contents

[**1.**  **Introduction** 3](#_Toc22213315)

[**2.** **Refraction and Visual Acuity** 3](#_Toc22213316)

[2.1 Visual Acuity Equipment and Facilities 3](#_Toc22213317)

[2.1.1 Introduction 3](#_Toc22213318)

[2.1.2 ETDRS Charts](#_Toc22213319) 3

[2.1.3 Retro-illuminated Standard ETDRS Light box 4](#_Toc22213320)

[2.1.4 Illumination 4](#_Toc22213321)

[2.1.5 4 meter and 1 meter Visual Acuity Lanes 4](#_Toc22213322)

[2.2 Refraction Technique 5](#_Toc22213323)

[2.2.1 Introduction 5](#_Toc22213324)

[2.2.2 Beginning Approximate Refraction 5](#_Toc22213325)

[2.2.3 Subjective Refraction 6](#_Toc22213326)

[2.2.4 Determination of Spherical component 6](#_Toc22213327)

[2.2.5 Determination of Cylindrical Refraction 8](#_Toc22213328)

[2.2.6 Refining Spherical Power 9](#_Toc22213329)

[2.2.7 Refraction for Subjects with Poor Initial Visual Acuity 10](#_Toc22213330)

[**3.** **Testing BCVA** 10](#_Toc22213331)

[3.1. 4 Meter Test 10](#_Toc22213332)

[3.2. 1 Meter Test 11](#_Toc22213333)

[3.2.1 Scoring BCVA 12](#_Toc22213334)

[3.3. Hand Motion Visual Acuity 12](#_Toc22213336)

[3.4. Light Perception & No Light Perception 13](#_Toc22213337)

[**Appendix 1: Refraction Protocol Summary** 14](#_Toc22213338)

[**Appendix 2: Visual Acuity Score Conversion** 15](#_Toc22213339)

# **1. Introduction**

This document details the procedures for conducting Refraction and Best Corrected Visual Acuity assessment. All examiners who will conduct Refraction and Best Corrected Visual Acuity examinations for subjects involved in trial XXX should be familiar with this manual and listed on the trial delegation log.

# **2. Refraction and Visual Acuity**

Refraction and Best Corrected Visual Acuity (BCVA) measurements are required for each eye at all time points given in the flow chart. All refraction and BCVA examinations are conducted prior to intraocular pressure measurement, pupillary dilation or anterior eye surface anaesthesia. Refraction will be performed using ETDRS Chart R only (not ETDRS Chart 1 or 2), using the equipment described in Section 2.1. Following refraction, visual acuity with the measured refraction will be assessed using ETDRS Chart 1 for the right eye and Chart 2 for the left eye. BCVA score is calculated as described in [Section 3.2.1](#S_3_2_1).

## **2.1 Visual Acuity Equipment and Facilities**

### 2.1.1 Introduction

Refraction and BCVA will be assessed utilizing a modified procedure adapted from the Early Treatment Diabetic Retinopathy Study (ETDRS). This procedure requires the following equipment:

- A standard ETDRS light box
- A set of three ETDRS Charts (Chart R, Chart 1 and Chart 2). Although discouraged, ETDRS charts with numbers or other symbols (Landolt C’s or Tumbling E’s) may be used for subjects who are unable to read letter charts
- Trial lens set (full aperture, wire rimmed, negative cylinder lenses)
- Jackson Cross Cylinders (JCC) (wire rimmed, ±0.25, ±0.50, ±1.00 D)
- Occluding lens
- Focimeter

BCVA examinations will be performed at 4 meters for all visits (or 1 meter if indicated).

### 2.1.2 ETDRS Charts

Chart R is used for refraction of both right and left eyes. Charts 1 and 2 are used for BCVA examination of the right and left eyes respectively. The chart features high contrast Sloan Letters in each of the 14 lines. The lines are of equal difficulty and a geometric progression of letter size (and therefore an arithmetic progression of the logarithm of minimum angle of resolution) from line to line. Charts R, 1, and 2 have different letter sequences. Subjects should be prevented from seeing Charts 1 and 2 until refraction has been completed and visual acuity assessment begins.

### 2.1.3 Retro-illuminated Standard ETDRS Light box

The dimensions of the light box are 62.9cm by 65.4 cm by 17.8 cm. The box can be mounted on a wall or on a stand.

Whether mounted on the stand or on the wall the light box should be mounted at a height such that the top of the third row of letters (0.8 logMAR) is 124 +/- 5.1 cm from the floor.

### 2.1.4 Illumination

All room lights should be turned off during refraction and BCVA assessments. Make the examination room as dark as possible as any additional light can adversely affect measurements. Ensure all blinds/curtains are drawn and doors shut. The light box, when switched on, should provide ample illumination for the examiner to record the test results (a small side lamp is permissible if required).

With the light box switched off, no more than 15 foot-candles of light should fall on the centre of the chart. These conditions should be reproduced at each study visit.

Light box illumination is provided by two Daylight 20-watt fluorescent tubes.

Each tube is partially covered by a 35.6 cm fenestrated sleeve, open at the back. The sleeve acts as a baffle to reduce illumination. Each sleeve should be centred on the tube such that an equal length of the tube, 10.6 cm, is left uncovered to the right and left of the sleeve. The opening at the backs of the sleeves should be positioned to point directly to the back of the box. The lower sleeve has a cut-out that should be pointed towards the ballast resistor. Newer tubes have fenestrated sleeves pre- attached to them therefore centration and orientation is not required.

As luminous output of fluorescent tubes diminishes by 5% during the first 100 hours and by another 5% during the next 2000 hours, new tubes should be kept “on” for about 96 hours before being used and all tubes should be replaced once a year. A log should be placed in the back of the light box with the date of the replacement of the bulbs duly signed.

### 2.1.5 4 meter and 1 meter Visual Acuity Lanes

Space Requirements

A distance of exactly 4 meters is required between the subject’s eyes and the chart in the light box for the 4 meter test. The examination room must have space for the 4 meter lane, light box (potentially with stand) and space for the seated subject.

The minimum space requirements vary according to how the box is mounted.

- Wall-mounted: 4 meter lane + 17.8 cm (depth of light box) + space for seated subject
- Stand-mounted: 4 meter lane + 33cm (two casters of the stand touching the rear wall, or a line on the floor if there is no wall) + space for the seated subject

Marking the Distance

4 meter distance:
The 4 meter examination location must be marked clearly in the examination room. The 1 meter examination location is not required to be marked. It must be measured during the examination using a 1 meter measuring device that should be rigid/non-flexible.

If the chair and the box are permanently affixed, distance measurements are only required to be made once.

If the box is mounted on a portable stand, the 4 meter distance must be marked clearly on the floor. The location and orientation of the box must be rechecked each time a chart is replaced or the box is touched. If the stand touches the rear wall of the room, two of the five casters should be in contact with the wall.

## 1 meter distance: This is measured from the eye of the subject, seated comfortably in the examination chair with their back firmly against the back of the chair to the front of the chart. This measurement must be taken using a non-flexible 1 meter measuring device. During 1 meter examinations care must be taken to ensure that this distance remains constant.

## **2.2 Refraction Technique**

### 2.2.1 Introduction

Chart R must be used for refraction. The right eye is refracted first and then the left eye. Refraction should always be attempted first at 4 meters. A 1 meter distance should only be used if a 4 meter refraction is not possible.

### 2.2.2 Beginning Approximate Refraction

At study visit 1 use the following options in order of decreasing preference:

1. The measured, prescribed or otherwise documented refractive power of the subject’s most current distance vision glasses
2. Results of retinoscopy or autorefraction
3. Results obtained from a non-study visit refraction
4. Refraction from plano

For all subsequent study visits (i.e. visit 2 onwards), the beginning approximate refraction will always be the final refraction result obtained from the previous study visit.

If the subject wears contact lenses, they should be advised to remove these on the day of the examination. If the subject presents wearing contact lenses, the contact lenses should be removed for a minimum of half an hour before any refraction or BCVA examinations commence. In this instance anterior eye assessment, particularly of the cornea, should be made using a slit lamp bio-microscope and any abnormalities should be recorded.

The refraction should be performed at 4 meters unless the subject is unable to read the 20/160 line at 4m with the beginning approximate refraction. In such cases refraction should be performed at a 1 meter distance with an addition +0.75 sphere added to the beginning approximate refraction to account for the reduced testing distance. If at 1 meter the subject is unable to read any letters from the top line of Chart R, a pinhole should be inserted. If an improvement in VA is noted with the pinhole, the reduction in vision can be attributed, in part, to refractive error and refraction should be performed. If there is no improvement with the pinhole then refraction is not indicated.

### 2.2.3 Subjective Refraction

The aim of subjective refraction is to determine the subject’s optimum refractive correction.

The trial frame should be fitted to the subject’s face such that the lens cells are parallel to the anterior plane of the orbits and centred in front of the pupils. For cases, in which both eyes are to be refracted it is conventional to refract the right eye first. The left eye is occluded by lightly patching with an eye pad or folded tissue with tape or by inserting the black occluding lens into the trial frame (at the examiner’s discretion). The beginning approximate refraction is placed in the right lens cells with the spherical lens in the posterior lens cell of the trial frame that is closest to the eye and the cylindrical correction in the cell immediately in front of the sphere. When refracting subjects with spherical corrections greater than or equal to 6 Diopters care should be taken to ensure that the spherical lens remains at a constant distance from the cornea.

The subject should be instructed to read the smallest legible line on Chart R. The subject should be encouraged to read the letters until no further meaningful responses are provided.

### 2.2.4 Determination of Spherical Component

Based on the initial visual acuity measured, the examiner will perform refraction using the lenses recommended in the Refraction Protocol Summary (see Appendix). It is important to note that if the subject’s visual acuity improves during the refraction process, different lens intervals may be required to be used. For example if the subject’s initial visual acuity is in the 20/100–20/160 range and improves to the 20/10-20/80 range the lenses used during the refraction must be altered to reflect this improvement. In the following example the initial visual acuity is between 20/10 and 20/80.

1. The subject is instructed to fixate upon the lowest legible line. A +0.50 sphere is then held in front of the right eye. The subject is asked if the vision is “better, worse or no different”. The examiner should use this exact phrase.
2. If the subject states that the vision is “better” or “no different” the sphere in the trial frame is replaced with one that is 0.50 diopters more positive/less negative. Confirm that the subject’s vision is at least as good as before addition of the +0.50DS lens. The +0.50 sphere should be held in front of the right eye again and the subject asked if the vision is “better, worse or no different”. This process of presenting the plus sphere is repeated until the subject says that the +0.50 sphere held in front of the trial frame makes the vision “worse”.
3. If the subject states that the vision is “worse” the examiner should remove the +0.50 sphere and proceed to step b.
4. A -0.37 sphere is held in front of the trial frame and the subject is asked if the vision is “better, worse or no different”.
5. If the subject states their vision is “better”, the examiner should ask the subject to try and read more letters. For minus power to be given, the subject must prove their vision is better by reading at least one additional letter on Chart R. If no additional letters are read no minus power should be given to the subject. If at least one additional letter is read, the sphere in the trial frame should be replaced with a sphere that is 0.25 diopters less positive/more negative. The -0.37 lens should then be shown to the subject again and the subject should be asked if the vision is “better, worse or no different”. This process should continue until the subject states that the vision is “worse”, “no different” or the subject is unable to read any further letters with the addition of more minus.
6. If the subject states that the vision is “worse”, “no different” or if the subject is unable to read any further letters, no further minus power should be added. The examiner should proceed to step c.
7. A +0.50 sphere is held in front of the right eye. The subject is asked if the vision is “better, worse or no different”. The examiner should use this exact phrase.
8. If the subject states that their vision is “better” or “no different” the sphere in the trial frame is replaced with one that is 0.50 diopters more positive/less negative. Confirm that the subject’s vision is at least as good as before addition of the +0.50DS lens. The +0.50 sphere should be held in front of the right eye again and the subject is asked if their vision is “better, worse or no different”. This process of presenting the plus sphere is repeated until the subject says the +0.50 sphere held in front of the trial frame makes the vision “worse”.
9. If the subject states that the vision is “worse” the examiner should proceed to determination of cylindrical refraction

### 2.2.5 Determination of Cylindrical Refraction

Both minus and plus- cylinder techniques are acceptable but for purposes of this discussion, only **plus-cylinder** technique techniques are presented

1. Cylinder Axis Determination.

If the beginning approximate refraction contains a cylindrical component, the cylinder axis should be assessed first. The choice of Jackson Cross Cylinder (JCC) is dependent upon the subject’s visual acuity (see Appendix 1). In this example a +/- 0.50 JCC is used (for VA 20/10-20/80).

The subject is instructed to fixate upon a single round letter (“C” or “O”) one or two lines above the smallest legible line on Chart R.

The subject is instructed: “I will show you two lenses. Tell me which lens makes the letter clearer, even if both blur the letter, or if the letter looks equally clear with both lenses”. The JCC is held by the examiner such that its handle is in line with axis of the cylinder in the trial frame. “Here is lens 1….”. The JCC is then flipped so that the positions of the positive and negative axes reverse, with the handle still in line with the cylinder axis “…and here is lens 2”.

If the subject finds one lens position clearer than the other, and the cylinder in the trial frame is plus, the cylinder is moved towards the positive axis of the JCC when held in the preferred position, in 1 to 10 degree steps (this is dependent upon the cylinder power, see Appendix 1). The process of showing the subject “lens 1” and “lens 2” continues until the subject notices no difference between the two lenses.

If the subject’s preferences move the axis first in one direction and then to the opposite direction, align the cylinder between the two preferred positions (bracketing).

If at the outset (with the first presentation of the JCC) the subject is unable to choose between “lens 1 or lens 2” the axis of the cylinder in the trial frame is moved 15 degrees in an arbitrary direction. The axis is checked in this new position with the JCC. If the subject’s choice indicates a return to the original axis, the cylinder in the trial frame is moved back to the original axis and the examiner should continue to determine cylinder power.

b. Cylinder Power Determination

Please refer to Appendix 1 for the choice of JCC power used, for this example we are using +/-0.25 JCC (for VA 20/10-20/80). Please note, the power of JCC required may change if the visual acuity improves. The subject is instructed to fixate upon a single round letter (“C” or “O”) one or two lines above the smallest legible line on Chart R. The subject is instructed: “I will show you two lenses. Tell me which lens makes the letter clearer, even if both blur the letter, or if the letter looks equally clear with both lenses”. The JCC is held by the examiner such that its positive axis is in line with axis of the cylinder in the trial frame. “Here is lens 1….”. The JCC is then flipped so that the position of the negative axis is in line with the axis of the cylinder in the trial frame “…and here is lens 2”.

If the subject prefers the positive axis of the JCC in line with the cylinder axis, the power of the cylinder in the trial frame is increased by +0.25 diopters. If the subject prefers the negative axis of the JCC in line with the cylinder axis, the power of the cylinder is decreased by 0.25 diopters. This process is repeated until the subject notices no difference between the two lens options.

For every 0.50 change in cylindrical power the spherical correction must be changed by 0.25 in the opposite power. For example, when using plus cylinders, a +0.50 increase in the cylindrical component of the refraction, will require a -0.25 spherical increase. Conversely, a +0.50 decrease in the cylindrical component of the refraction, will require a +0.25 spherical increase.

If the subject’s response indicates a change that would require removal of the cylinder, the examiner should test for cylindrical power at an axis 90 degrees from the previous axis.

If the beginning approximate refraction contains no cylinder (“pure” sphere), the presence of astigmatism should be tested using the following method:

The 0.50 JCC is placed with the positive axis at 90 degrees. The subject is asked which lens is preferred. “1” with the positive axis at 90 degrees or “2” (the JCC is flipped) with the positive axis at 180 degrees. If neither of the two lenses is preferred, the JCC is placed with the positive axis at 45 degrees. The subject is asked whether “lens 3”, with the positive axis at 45 degrees or “lens 4” with the positive axis at 135 degrees is preferred. If the subject prefers any of the 4 lenses a +0.50 cylinder is placed at the preferred axis. The axis should be checked and then the cylinder power in the manner described above.

### 2.2.6 Refining Spherical Power

The power of the sphere is refined by challenging the subject with +0.25 and -0.37 spheres and changing the power of the sphere as necessary, as described in [Section 2.2.4](#_2.2.4_Determination_of). It is important to note that the lenses used to challenge the subject and the changes made to the refraction during the refinement differ to those used when determining Best Vision Sphere. The Refraction Protocol Summary (see Appendix) provides guidance on the correct lenses to be used for specific visual acuity ranges.

If the final refraction result differs from the beginning approximate refraction by 2.00 diopters or more, it is imperative that the subject is able to read just as well as with the beginning approximate refraction. If this is not the case the refraction procedure must be repeated.

The Refraction result obtained for the right eye is recorded on the Refraction Worksheet. The entire process is repeated for the left eye.

### 2.2.7 Refraction for Subjects with Poor Initial Visual Acuity

If it is not possible for the subject to read the 20/160 at 4 meters, the refraction should be performed at 1 meter. Prior to refracting at 1 meter a +0.75 sphere should be added to the trial frame to account for the closer distance. The 1 meter distance must be verified using a non-flexible measuring device. If subjective refraction can be performed successfully at 1 meter, the +0.75 sphere should be subtracted from the final refraction result to make the correction appropriate for the 4 meter distance. This correction should be recorded on the Refraction Worksheet. (Reminder: Visual acuity will be tested first at 4 meters even if the subject is refracted at 1 meter). If, during refraction at 1m, the vision improves to the extent that a 4m refraction is possible, move the subject to 4m and refract at this distance.

**3. Testing BCVA**

- Testing of BCVA must begin at 4 meters
- If the subject is unable to read 20 letters or more at 4 meters, the BCVA test should then be performed at 1 meter

## **3.1. 4 Meter Test**

Chart 1 is used to test the subject’s right eye. The left eye should be occluded. Following this the subject’s left eye is tested with Chart 2 and the right eye occluded. Each chart should remain hidden from view until the eye in question is ready for testing.

The subject should be seated at 4 meters with their back firmly against the back of the chair. The examiner should ensure that the subject is seated comfortably, the subject’s head must not move forward or backward during the test, although eccentric fixation is encouraged.

Testing BCVA is not a test of intelligence, memory, concentration nor ability to remember or follow instructions, although all of these factors are involved in the test. The subject should be told that the chart has five letters and no numbers in each line. If the subject reads a number instead of a letter, they should be reminded that the chart only contains letters and encouraged to offer an alternative letter.

The subject should be asked to read the chart slowly (at a rate not faster than one letter per second) as this helps to achieve the best identification of each letter. The subject should not proceed to the next letter until they have given a definite response to the current letter. It is sometimes useful to demonstrate the letter per second pace to the subject by reciting “A…B…C….”. If at any stage the subject reads too quickly, they should be asked to stop and reminded to read slowly. If the subject loses their place, the examiner should ask the subject to go back to the first letter of the line before getting lost. Examiners should never point to the chart or to specific letters or numbers. Nor should the examiner read any of the letters during the test. A white sheet of paper may be used to guide the subject to the proper location on the chart.

Letters read correctly should be circled. Those letters read incorrectly should be marked with an ‘X’ and un-attempted letters should be left unmarked. Once a subject has identified a letter with a definite single letter response and has read the next letter, a correction of the previous letter cannot be accepted. If the subject changes a response **before** they have read the next letter in the sequence, the change **should** be accepted.

When a subject says they cannot read a letter, they should be encouraged to guess. If the subject gives more than one responses to a letter, they should be asked to choose one letter and if necessary to guess.

When the subject reads four or more letters on a line incorrectly, the examiner should stop the test for that eye.

There are several reasons to encourage the subject to guess:

- Subject’s statements that they cannot identify a letter are often unreliable
- Encouraging a subject to guess helps to maximise the subject’s effort
- It helps to assure uniformity among procedures performed at different sites
- It may help to prevent subject bias

## **3.2. 1 Meter Test**

If the subject reads fewer than 20 letters at 4 meters, visual acuity must be tested at 1 meter. It is advisable to obscure the chart from view when moving from 4 meters to 1 meter. When refracting or measuring BCVA at 1 m the fellow eye should be occluded by lightly patching with an eye pad or folded tissue with tape and by inserting the black occluding lens into the trial frame.

Before commencing the test at 1 meter a +0.75 sphere should be added to the 4 meter correction to compensate for the closer testing distance. The avoidance of any head movement is particularly important during the 1 meter test. The subject should be asked to read the first 6 lines only in the same manner as described in Section 3.1.

After right eye testing is completed, occlusion should be changed from left to right eye and Chart 1 replaced with Chart 2. The test is repeated for the left eye, starting at 4 meters.

### 3.2.1 Scoring BCVA

On the Best Corrected Visual Acuity Worksheet, all letters read correctly should be circled. Letters read incorrectly should be marked with an ‘X’ and letters not attempted should be left unmarked. The total number of letters read correctly should be recorded. Every box/underline should be filled out. If visual acuity was not tested at 1 meter and if hand movements and light perception testing was not required both sections should be crossed out.

The visual acuity score is calculated as follows:

If BCVA is tested at 4 meters and 20 or more letters read:

BCVA Score = the number of letters read correctly at 4 meters + 30

If BCVA is tested at 4 meters and fewer than 20 letters read

BCVA Score = the number of letters read correctly at 4m + the number of letters read correctly at 1m

If BCVA is tested at 1 meter, when no letters are read correctly at 4m:

BCVA Score = the number of letters read correctly at 1 meter

If no letters are read correctly at 4 meters or 1 meter:

BCVA score is 0 and the subject should be examined using Hand Movements and Light perception. See sections 3.3 and 3.4 respectively.

## **3.3. Hand Motion Visual Acuity**

The examiner’s hand with all fingers spread out should be extended 2 feet (61 cm) directly in front of the eye being examined. All room illumination should be switched on.

The fellow eye must be occluded with an occluding lens and eye pad or tissue with tape. A light should be shone directly on the hand from behind the subject. The examiner’s hand should be moved up and down (vertically) or side to side (horizontally) at a constant speed of approximately one back and forth presentation per second. The subject is instructed that the examiner’s hand will be presented and they will have to respond to the request: “Tell me when my hand moves and in which direction: up and down or side to side”. This should be repeated five times. Four out of five correct responses indicate that hand motion vision is present. If the subject does not correctly identify hand motions in four of the five trials then light perception must be tested.

## **3.4. Light Perception & No Light Perception**

Light perception should be tested with an indirect ophthalmoscope or retinoscope in a darkened room. The indirect ophthalmoscope light should be in focus at 3 feet (90 cm) with the rheostat set at maximum voltage. From that distance, the beam should be directed in and out of the pupil of the eye being examined at least four times. The subject should be asked to indicate when they see the light. If the examiner is convinced that the subject perceives the light, vision should be recorded as “light perception” if not, vision should be recorded as “no light perception”.

# **Appendix 1: Refraction Protocol Summary**

| **Vision with Beginning Approximate Refraction at 4 meters** | **Sphere** | | **Cylinder Axis** | **Cylinder Power** | | **Sphere Refinement** | |
| --- | --- | --- | --- | --- | --- | --- | --- |
|  | **Sphere for Checking** | **Incremental Sphere Power Change** | **JCC for Checking** | **JCC for Checking** | **Incremental Cylinder Power**  **Change** | **Sphere Power for Checking** | **Incremental Sphere Power**  **Change** |
| 20/10 – 20/80 | +0.50 | +0.50 | 0.50 JCC | 0.25 JCC | +0.25 | +0.25 | +0.25 |
| (refract at 4 | -0.37 | -0.25 |  |  | or | -0.37 | -0.25 |
| meters) | +0.50 | +0.50 |  |  | -0.25 | +0.25 | +0.25 |
| 20/100 – | +1.00 | +1.00 | 1.00 JCC | 1.00 JCC | +1.00 | +0.50 | +0.50 |
| 20/160 | -1.00 | -1.00 |  |  | or | -0.37 | -0.50 |
| (refract at 4 | +1.00 | +1.00 |  |  | -1.00 | +0.50 | +0.50 |
| meters) |  |  |  |  |  |  |  |
| 20/200 – | +2.00 | +1.00 | 1.00 JCC | 1.00 JCC | +1.00 | +1.00 | +1.00 |
| 20/400 | -2.00 | -1.00 |  |  | or | -1.00 | -1.00 |
| (refract at 1 | +2.00 | +1.00 |  |  | -1.00 | +1.00 | +1.00 |
| meter) |  |  |  |  |  |  |  |
| < 20/400 | +2.00 | +1.00 | No cylinder test | | | No refinement | |
| (refract at 1 | -2.00 | -1.00 |  | | |  | |
| meter) | +2.00 | +1.00 |  | | |  | |

**Axis Step Sizes for Refinement of Cylinder**

| **Cylinder Power** | **Axis Step Size** |
| --- | --- |
| < 1.00 D | 10^o^ |
| 1.00 - < 2.00 D | 5 ^o^ |
| 2.00 - < 3.00 D | 3 ^o^ |
| 3.00 - < 5.00 D | 2 ^o^ |
| 5.00 - < 8.00 D | 1 ^o^ |

# **Appendix 2: Visual Acuity Score Conversion (for reference)**

| **Letter Score** | **logMAR** | **Snellen Equivalent** |
| --- | --- | --- |
| 1 to 5 | 1.68-1.60 | 20/800 |
| 6 to 10 | 1.58-1.50 | 20/640 |
| 11 to 15 | 1.48-1.40 | 20/500 |
| 16 to 20 | 1.38-1.30 | 20/400 |
| 21 to 25 | 1.28-1.20 | 20/320 |
| 26 to 30 | 1.18-1.10 | 20/250 |
| 31 to 35 | 1.08-1.00 | 20/200 |
| 36 to 40 | 0.98-0.90 | 20/160 |
| 41 to 45 | 0.88-0.80 | 20/125 |
| 46 to 50 | 0.78-0.70 | 20/100 |
| 51 to 55 | 0.68-0.60 | 20/80 |
| 56 to 60 | 0.58-0.50 | 20/63 |
| 61 to 65 | 0.48-0.40 | 20/50 |
| 66 to 70 | 0.38-0.30 | 20/40 |
| 71 to 75 | 0.28-0.20 | 20/32 |
| 76 to 80 | 0.18-0.10 | 20/25 |
| 81 to 85 | 0.08-0.00 | 20/20 |
| 86 to 90 | -0.02 to-0.08 | 20/16 |
| 91 to 95 | -0.12 to -0.18 | 20/12.5 |
| 96 - 100 | -0.22 to -0.28 | 20/10 |
